# Web-based Medical Education During COVID-19 Lockdown: A Step Back or a Leap to the Future?

**DOI:** 10.1177/15347346211011848

**Published:** 2022-09

**Authors:** Anna Eleftheriou, Aikaterini Rokou, Christos Argyriou, Nikolaos Papanas, George S. Georgiadis

**Affiliations:** 1 Medical School, 37791 Democritus University of Thrace, Alexandroupolis, Greece; 2 37791 Department of Vascular Surgery, Democritus University of Thrace, Alexandroupolis, Greece; 3 Diabetes Centre, 387479 Democritus University of Thrace, Alexandroupolis, Greece

**Keywords:** COVID-19, medical education, students, telemedicine

## Abstract

The impact of coronavirus infectious disease (COVID-19) on medical education has been substantial. Medical students require considerable clinical exposure. However, due to the risk of COVID-19, the majority of medical schools globally have discontinued their normal activities. The strengths of virtual teaching now include a variety of web-based resources. New interactive forms of virtual teaching are being developed to enable students to interact with patients from their homes. Conversely, students have received decreased clinical training in certain medical and surgical specialities, which may, in turn, reduce their performance, confidence, and abilities as future physicians. We sought to analyze the effect of telemedicine on the quality of medical education in this new emerging era and highlight the benefits and drawbacks of web-based medical training in building up future physicians. The COVID-19 pandemic has posed an unparalleled challenge to medical schools, which are aiming to deliver quality education to students virtually, balancing between evidence-based and experience-based medicine.

## Editorial

The coronavirus infectious disease (COVID-19) pandemic has brought about drastic changes in medical communities worldwide, altering the delivery of care to patients, reducing medical and surgical patient exposure to hospitals, and disrupting “traditional” programs of medical education. Although the era of vaccination has initiated, social distancing and quarantine are still the only effective and widely available interventions.^1^ For this reason, medical societies globally have issued recommendations that exposure of medical students to direct patient care activities should be avoided at all costs.^2^ Although the surge of telemedicine will revolutionize medical education, enabling medical students to further explore and upgrade their current technological assets, innovative methods and practices should be implemented in order to maintain a high quality of education. Indeed, this abrupt transition to distance learning comes at the cost of physical, emotional, and academic involvement, while the potential long-term effects of this “education crisis” need to be evaluated.^1,2^

An important element of telemedicine, especially in senior medical students, is virtual clinical teaching, which has been shown to be one of the most effective forms of teaching.^3,4^ Virtual teaching enables medical students to gain the benefits of engagement, patient interaction, and feedback from tutors, while eliminating any risk of infection from physical presence in COVID-19 patient wards or rotation in medical facilities.^3,4^ This mode of teaching has met with student satisfaction, augmenting their self-confidence, clinical reasoning, and communication skills.^5,6^ Conversely, virtual teaching has been criticized for lack of establishing clinical connections, physical contact with the patient, and competitiveness among students.^7^ Loss of competitiveness is especially crucial in clinical-grade medical students, who can possibly lag behind in surgical and clinical skills.^8^ Thus, impaired motivation and loss of their future role as specialized physicians may occur.^8^

Active participation and education of medical students could be redirected to alternative methods of involvement.^5^ Students at Harvard Medical School have developed student-led virtual education and activism committees consisting of 500 students overall, for both health care professionals and the community.^5^ These committees are responsible for providing educational material for nonmedical communities and learning resources for medically literate audiences.^5^ The activism group has responded to clinical needs from frontline health care workers by recruiting students for specific tasks based on their level of training and research assistance through the creation of COVID-19 safety protocols and clinical guidelines.^5^ Although it may appear impressive to talk about online or virtual education training, according to Sahi et al,^9^ these modalities require trained manpower, healthy finances, and improvements in infrastructure and technology. Accordingly, virtual medical teaching is time demanding and expensive, limiting its implication in low-income countries.^9^

The acquisition of basic medical skills, such as physical examination and history taking, are key components of a successful training for junior and preclinical medical students. Teaching programs have documented their efficacy in increasing students’ confidence in their skills and medical abilities.^10^ Furthermore, “hybrid” techniques involving traditional in-person learning combined with synchronous or asynchronous e-learning have demonstrated a marked improvement in history taking, practical training, and surgical performance.^11^ For example, Machado et al^12^ suggested the “inverted classroom method,” a blended-learning method where the self-directed learning phase precedes the classroom-instruction phase. However, in an exclusively online training program, medical students would probably have to rely on purely theoretical knowledge, augmenting their dependency on radiographic imaging rather than actual physical contact with the patient and clinical skills.^12^ The risk of delayed or wrong diagnosis is there.

Future physicians should be able to initiate primary management of a medical emergency, which is essential in minimizing morbidity and mortality.^1-4^ This highlights the need to provide more opportunity for practical, hands-on teaching and emergency simulations. Certainly, no role playing can reliably simulate the pressure of a “real-life” medical condition or emergency. Nonetheless, participation in a practical training course may significantly enhance students’ self-confidence in managing emergencies and accomplishing specific tasks.^13^ Still, some forms of assessments, such as laboratory practical assessments, are impossible to conduct via the internet.^14^ The transition from face-to-face teaching to online delivery has a serious impact on assessments and evaluation. Moreover, such an approach should be implemented gradually from the first years of medical school as an adjunct to the traditional medical training. This rapid and unexpected change of circumstances and loss of structure can lead to increased social withdrawal, contributing to students’ anxiety and loneliness.^15^

A direct comparison between e-learning and traditional teaching is difficult because there are no objective measures to assess effectiveness. Currently, whether simulation training is enough or an effective adjunct to hands-on training is debated. Even before the first case of COVID-19 was diagnosed, technological innovation had already begun to change education, health care, and even social relationships.^16^ The COVID-19 crisis has simply accelerated the drive and interest in these new tools. Despite the innovations of virtual teaching, available data are restricted by the small number of participants and the few medical faculties implementing these educational innovations. Medical training in the COVID-19 era remains crucial, and we should do our best to implement, evaluate, and improve new virtual teaching tools. The latter are needed to develop future clinicians and future researchers.^17,18^
